# Pathogen Spillover to an Invasive Tick Species: First Detection of Bourbon Virus in *Haemaphysalis longicornis* in the United States

**DOI:** 10.3390/pathogens11040454

**Published:** 2022-04-10

**Authors:** Alexandra N. Cumbie, Rebecca N. Trimble, Gillian Eastwood

**Affiliations:** 1 Department of Entomology, College of Agriculture and Life Sciences, Virginia Polytechnic Institute and State University (Virginia Tech), Blacksburg, VA 24061, USA; 2 Department of Biochemistry, Virginia Polytechnic Institute and State University (Virginia Tech), Blacksburg, VA 24061, USA; trebecca@vt.edu; 3 Center for Emerging Zoonotic and Arthropod-Borne Pathogens (CeZAP), Virginia Polytechnic Institute and State University (Virginia Tech), Blacksburg, VA 24061, USA; 4 The Global Change Center, Virginia Polytechnic Institute and State University (Virginia Tech), Blacksburg, VA 24061, USA

**Keywords:** Asian longhorned tick, Bourbon virus, *Haemaphysalis longicornis*, pathogen spillover

## Abstract

*Haemaphysalis longicornis* (Neumann, 1901) (Acari: Ixodidae), the Asian longhorned tick, is an invasive tick species present in the USA since at least 2017 and has been detected in one-third of Virginia counties. While this species is associated with the transmission of multiple pathogens in its native geographical range of eastern Asia, little is known about its ability to acquire and transmit pathogens in the USA, specifically those that are transmissible to humans, although from an animal health perspective, it has already been shown to vector *Theileria orientalis* Ikeda strains. Emerging tick-borne viruses such as Bourbon virus (genus: *Thogotovirus*) are of concern, as these newly discovered pathogenic agents have caused fatal clinical cases, and little is known about their distribution or enzootic maintenance. This study examined *H. longicornis* collected within Virginia (from ten counties) for Bourbon and Heartland viruses using PCR methods. All ticks tested negative for Heartland virus via qRT-PCR (S segment target). Bourbon-virus-positive samples were confirmed on two different gene targets and with Sanger sequencing of the PB2 (segment 1) gene. Bourbon virus RNA was detected in one nymphal stage *H. longicornis* from Patrick County, one nymph from Staunton City, and one larval pool and one adult female tick from Wythe County, Virginia. An additional 100 *Amblyomma americanum* (Linnaeus 1758; lone star tick) collected at the same Patrick County site revealed one positive nymphal pool, suggesting that Bourbon virus may have spilled over from the native vector, potentially by co-feeding on a shared Bourbon-virus-infected vertebrate host. Blood tested from local harvested deer revealed a 11.1% antibody seroprevalence against Bourbon virus, exposure which further corroborates that this tick-borne virus is circulating in the southwest Virginia region. Through these results, it can be concluded that *H. longicornis* can carry Bourbon virus and that pathogen spillover may occur from native to invasive tick species.

## 1. Introduction

Bourbon virus (BRBV) is a vector-borne agent from the genus *Thogotovirus*, family *Orthomyxoviridae*, associated with clinical disease cases that were initially identified in Kansas, Oklahoma, and Missouri in 2014, 2015, and 2017, respectively [1,2,3]. Thogotoviruses are enveloped, segmented, negative-sense RNA viruses transmitted from arthropods, including ticks [4,5]. Prior to 2014, the only strains of thogotoviruses globally characterized as human pathogens were Dhori virus and Thogoto virus. Dhori virus was first isolated from *Hyalomma dromedarii* (Koch, 1844) in 1973 [6] and was identified as a human pathogen in 1987 following accidental lab exposure events [7,8]. Thogoto virus was first identified as a human pathogen in two African patients in 1966 causing febrile illness, meningitis, and neurological sequelae [9]. Although Aransas Bay virus is a thogotovirus in the USA, it is not associated with human illness [1]. In 2014 however, a novel Thogotovirus (related to Dhori virus) was isolated from a fatal case in Bourbon County, Kansas, USA [1,10]. The discovery of BRBV was the first case of human pathogenic Thogotovirus infection in North America, and the viral isolate was named Bourbon virus strain Original (BRBV-KS). To date, there have been two additional cases of human BRBV infections in the USA: (i) a resident in Oklahoma who became infected and recovered in 2015 and (ii) a fatal case of BRBV infection in an immunocompromised individual in Missouri, USA, in 2017 [2,3].

Another emerging arbovirus of medical importance in the USA is Heartland virus (HRTV). Heartland virus is a tick-borne phlebovirus from the family *Phenuviridae*, genus *Bandavirus*, first identified in 2009 when it was isolated from two clinical cases in Missouri [11]. Closely related to severe fever with thrombocytopenia syndrome virus (SFTSV), HRTV is a bunyavirus that has been isolated in residents of various Asian countries, including China, Japan, and Korea, and which is associated with the tick *Haemaphysalis longicornis* in its native range [12,13,14]. Currently, HRTV is responsible for over 50 clinical cases in the USA, primarily in the Midwest and South [15]. Heartland virus can cause leukopenia, thrombocytopenia, and multiple organ failure in infected individuals and has been associated in the deaths of at least three people [16,17,18].

The most probable route of transmission for BRBV and HRTV to humans is from the bite of an infected *Amblyomma americanum* L. tick [19,20,21]. Aside from its detection and characterization in human cases, serological evidence of BRBV and HRTV has been reported in wildlife sera [22,23,24,25] and in field-collected *A. americanum* [2,19,21,26].

The Commonwealth State of Virginia, USA, is home to a variety of tick species, including ticks of medical and veterinary importance—*Ixodes scapularis* (Say, 1821), *Dermacentor variabilis* (Say, 1821), *A. americanum*, and, more recently, *H. longicornis*, the Asian longhorned tick. *Haemaphysalis longicornis* is an invasive tick species in Virginia and across the eastern USA, following first reports of the species on a sheep in New Jersey in 2017 [27]. Populations of *H. longicornis* have since been reported in 17 states, including 38 counties in Virginia as of March 2022 [28]. In its native range of East Asia, *H. longicornis* is a vector for multiple bacterial, viral, and protozoan pathogens, and thus, its arrival in the USA represents a huge concern for public and animal health. Research efforts are focused on understanding the threat it poses as a vector of existing or novel tick-borne pathogens within the USA. In western Virginia, incidence of vector-borne disease is increasing [29,30], including the movement and transmission of emerging tick-borne viruses [31]. The presence of sympatric populations of *I. scapularis, A. americanum*, and *D. variabilis* with *H. longicornis* creates an opportunity for pathogen spillover through co-feeding and sharing of hosts. *Haemaphysalis longicornis* is already responsible for the transmission of *Theileria orientalis* Ikeda strain to cattle in Virginia [32,33]. Additionally, it has been demonstrated that laboratory-raised *H. longicornis* can acquire and transmit *Rickettsia rickettsii*, the causative agent of Rocky Mountain spotted fever [34]. More recently, it has been reported that field-collected *H. longicornis* can harbor *Anaplasma phagocytophilum*, the causative agent of human granulocytic anaplasmosis [35].

The purpose of this study was to screen local *H. longicornis* populations for emerging tick-borne viruses in Virginia, specifically BRBV and HRTV. Although there have been neither reports of human cases of BRBV or HRTV infection in Virginia nor previous detections of the virus in ticks in the region, it was hypothesized that BRBV and HRTV were circulating in Virginia. Supportive of that notion are preliminary serological studies conducted in our laboratory revealing the presence of BRBV and HRTV antibodies in wildlife of northern Virginia, including striped skunks, white-tailed deer, and northern raccoons, among others [36]. In this study, we report the first detection of BRBV in *H. longicornis* ticks in the USA as well as the first detection of BRBV-infected ticks in Virginia.

## 2. Results

### 2.1. Haemaphysalis longicornis Collections in Western Virginia

A total of 1769 *H. longicornis* (598 larvae, 856 nymphs, and 315 females) were collected from sites across western Virginia in 2021 (Table 1). Due to limits on resources and the storage condition of some of the samples, a subset of 636 ticks (441 larvae, 153 nymphs, 42 adults) tested initially as 34 pools were chosen for the pathogen screening reported in the current study. All ticks collected from Fauquier, Floyd, Montgomery, Patrick, Staunton, Rockbridge, Roanoke, and Warren Counties were tested for viral pathogens as described. Larvae collected in Pulaski County had not been cryofrozen and thus were not tested for viral pathogens here. Due to high volumes of nymphs and adults collected from Wythe County, only a subset of ticks from those sites were tested for viral pathogens (126 larvae, 42 nymphs, and 24 adult females).

### 2.2. Arbovirus Detection in Ticks Utilizing Real-Time RT-PCR

A total of 34 tick pools (*n* = 636 ticks) were tested for BRBV and HRTV using real-time RT-PCR. No pools were positive for HRTV via the probe-based RT-PCR assay. Of the 34 tick pools, 4 (11.8%) were found potentially positive for BRBV using the SYBR Green RT-PCR assay amplifying a portion of the PB1 (segment 2) gene (Table 2). We detected BRBV-positive *H. longicornis* tick pools from three of 10 counties surveyed in western Virginia. BRBV-positive pools included one nymph from Patrick County, one nymph from a pool of 10 from Staunton City County, and a larval pool (*n* = 125) and one adult female from a pool of 8 from two separate sites in Wythe County. Across all 10 counties, the minimum infection rate (MIR) was calculated based on the number of positive pools divided by the total number of ticks yielding a minimum infection percentage of 0.62%. Only one nymph was collected in Patrick County and 15 ticks in Staunton City, and thus, no MIR was calculated for these counties, but a minimum infection percentage for Wythe County was calculated as 1.04% of ticks tested as being positive for BRBV. By subsequently examining individual ticks that had comprised each positive pool, one female from the pool of 8 adults from Wythe County was found to be BRBV-positive, along with one nymph from the pools of 10 nymphs from Staunton.

A subset of *A. americanum* (*n* = 100) collected at the same site as the BRBV-positive *H. longicornis* nymph in Patrick County were also tested for BRBV and HRTV. Seventy-one nymphal *A. americanum* ticks were tested as 12 nymphal pools (up to nine ticks per pool), and 29 adult ticks were tested individually. Evidence of BRBV was detected in 1 nymphal pool with a C(q) value of 34.98. Unfortunately, we were unable to obtain a sequence amplicon from this pool.

### 2.3. Virus Confirmation and Sequence Analysis

Each BRBV-positive tick pool or any BRBV-positive individual ticks from those pools were confirmed for viral evidence using Sanger sequencing. For each sample, we attempted to amplify two fragments from the PB2 gene (357 bp and 152 bp). The amplicons that were purified and sequenced were the direct products of the real-time RT-PCR assays. Amplicons from the single nymph in the pool from Patrick County, a single nymph from the Staunton City nymphal pool (*n* = 10), the larval pool from Wythe County, and a single female tick from the Wythe County pool (*n* = 8) were successfully sequenced in both directions for the larger PB2 (segment 1) gene fragment (Table 3). Amplicons for each of the potential BRBV-positive tick pools matched most closely to known isolates of Dhori thogotovirus strain Bourbon virus (GenBank accession no. MT628410) and other various isolates of Bourbon virus (accession nos. MK453529 and KU708253). These positive tick pools came from 3 different counties, each geographically distinct from the other.

### 2.4. Tick Homogenate Inoculation on Vero-76 Cells for Attempted BRBV Culturing

None of the tick homogenates inoculated onto Vero cell culture of the four BRBV qPCR-positive samples had displayed CPE by day 8. Despite a subsequent blind passage of each supernatant onto a fresh Vero cell monolayer for a further 7 days monitoring, no additional visualization of CPE was revealed, and RNA extracted from each supernatant failed to detect evidence of BRBV during qRT-PCR assay on these passaged supernatants.

### 2.5. Wildlife Serology

Wildlife blood samples (*n* = 93) collected in 2021 were screened for antibody evidence of exposure to BRBV. Nine white-tailed deer (of eighty-one tested), one raccoon (of four tested), and three groundhogs (of five tested) showed neutralizing antibodies against BRBV (PRNT_80_-positive at a minimum sera dilution of 1:20, end-point titers shown in Table 4). No evidence of exposure to BRBV was suggested from the single eastern cottontail or two striped-skunks tested. Bourbon virus seroprevalence rate in local white-tailed deer was thus 11.1% (95% CI: 4.3, 18.0).

## 3. Discussion

We report the first evidence of BRBV in local *H. longicornis* populations and wildlife in Virginia. Moreover, we report the first evidence of BRBV in a new tick species, *H. longicornis*, an invasive tick which is of veterinary and public health importance.

*Haemaphysalis longicornis* was first detected in the USA in 2017 and has since been detected on 52 avian species [37] and over 36 mammalian species in the country [38,39]. This tick species has already vectored a new pathogen to livestock in the USA, *T. orientalis* Ikeda genotype. It is detected in environments that house sympatric local tick species, such as *A. americanum* and *D. variabilis*, allowing for the potential of pathogen spillover. The spillover of BRBV into *H. longicornis* raises public health concern although it remains to be determined if the tick is also a competent vector and whether this virus can be transmitted onwards to subsequent hosts. Bourbon virus from *H. longicornis* could be a new strain with different infection and transmission dynamics in this species. We emphasize the need for disease pathogen surveillance anywhere that the tick is present.

We corroborate evidence that BRBV is now circulating in multiple counties in western Virginia through detection of the virus in locally collected ticks and BRBV-seropositive white-tailed deer and groundhogs. Although seropositivity does not indicate a reservoir host role, it does indicate exposure to an infectious agent; serosurveys of wild and domestic animal species as well as continued vector surveillance can demonstrate the introduction and expansion of novel arboviral pathogens. The three counties from which BRBV-seropositive wildlife were detected (Floyd, Roanoke, and Montgomery County) border or are close to Wythe and Patrick County, where BRBV-positive ticks were identified in the current study. Patrick County is at the southern border of Virginia adjacent to North Carolina, a state where harvested deer in two counties have shown BRBV-seroprevalence albeit at a higher reported rate than in this study (56%, *n* = 32) [23]. Even if geographically distant, deer or other wildlife hosts could become infected and/or also distribute infected ticks within their home ranges or into larger areas. The abundance and distribution of potential vertebrate host species are important to understand the movement and transmission of pathogens like BRBV and HRTV. Seroprevalence studies to determine infection exposure to both viral pathogens, based on larger sample sizes and additional wildlife species, are ongoing.

Potentially, the BRBV-infected ticks detected in this study could have fed on an infected vertebrate host or co-fed with infected ticks on the same host. Our site in Patrick County, for example, is inundated with *A. americanum* ticks, highlighting the potential for pathogen spillover through co-feeding or sharing of hosts as a likely transmission route for BRBV to the nymph. If BRBV is circulating in Virginia (albeit at low levels) in wildlife hosts or *A. americanum* ticks, these interactions are the suggested means of which *H. longicornis* in Patrick, Wythe, and Staunton counties are acquiring BRBV. Each of these counties has reported established *A. americanum* and white-tailed deer (the key vertebrate host of adult *A. americanum* and a species upon which *H. longicornis* have been reported) populations [28,40]. Future work aims to assess the pathogen flow and ecology of BRBV at these sites.

Transovarial transmission can be ecologically significant for the persistence of several arboviruses, e.g., La Crosse virus [41]; however, it generally occurs at low rates depending on the virus in question. Vertical transmission has been suggested to occur in the native tick vector, *A. americanum,* for both Heartland and Bourbon virus [20,42]. It is more likely for arboviruses to be maintained (i) horizontally in transmission between vector and host, (ii) through co-feeding adjacent to an infected tick on a host, or (iii) transstadially between life stages. Nevertheless, the detection of BRBV-infected larvae in our study indicates that *H. longicornis* ticks may acquire BRBV through transovarial transmission. Thus far, no field-collected larvae in the USA have been detected with BRBV in environmental surveillance sampling. Experimentally infected *A. americanum* larval ticks showed transstadial transmission of BRBV and a low rate of vertical transmission from infected female ticks [20]. In a laboratory study using *Rhipicephalus appendiculatus*, Thogoto virus was reported to be transmitted transstadially but not transovarially [43].

The prevalence rate of BRBV in field-collected *A. americanum* is described as 3.1% for nymphs and 3.2% for adult males in Missouri [2] and 2.5% for adults in Kansas [19] based on calculated maximum likelihood estimates (MLE). In our study, since pool size varied, we calculated MIR instead of using MLE. We detected a minimum infection percentage of 0.62% across all *H. longicornis* sampled (albeit based on a lower sample size and, here, including larvae).

Being able to isolate the strain(s) circulating in local populations and characterize their disease dynamics will provide a better idea of the risk this poses to local residents, domestic animals, and wildlife in counties with infected *H. longicornis*. Unfortunately, culturing of molecularly positive tick homogenates on Vero-76 cells in this study did not establish a novel isolate of BRBV. Potentially the strains of BRBV that we detected in *H. longicornis* ticks have a different phenotype, or potentially, culturing and sequencing was challenged due to low concentrations of viral particles in the tick specimens. It is possible that *H. longicornis* is not a competent vector or amplifying host for BRBV transmission as compared to *A. americanum*.

Nevertheless, these findings influence future studies of BRBV surveillance by highlighting an additional tick species that could be playing a role in the enzootic cycle of BRBV. Future studies of the transmission dynamics of BRBV in both *A. americanum* and *H. longicornis* would be beneficial in understanding the disease ecology of this emerging pathogen in the USA.

While no evidence of HRTV was detected during the current study, future research is needed to establish whether this virus is circulating with an underlying presence in the Virginia region and if any risk of pathogen spillover to different tick vectors exists in general. Although previous studies did not find serological evidence of HRTV in Virginia, this phlebovirus is suggested to be widespread in the eastern USA [25]. Indeed, both human cases and antibodies against HRTV in wildlife have been described in neighboring Tennessee and North Carolina. In New York State, HRTV circulation was evidenced via seroconversion in people, deer serosurveys, and HRTV detection from *A. americanum* [44]. The potential for spillover of HRTV into *H. longicornis*, which also exists in the northeastern USA, has yet to be seen.

The rapidly expanding distribution of *H. longicornis* and an ability to acquire BRBV and other pathogens of human disease within the USA [34,35] raise a concern for the spread and potential enzootic transmission of such disease agents. If *H. longicornis* is a competent vector of viral pathogens, then people living in areas with large populations of this tick species would be at an increased risk for encountering an infected tick, as *H. longicornis* has been reported to bite humans [45]. Given that it is frequently sympatric with the native BRBV vector *A. americanum*, future spillover events are not impossible.

Bourbon virus is a recently identified human-pathogenic thogotovirus which has been exclusively detected in the USA. Although human disease cases are rare and have so far been restricted in occurrence in the Midwest (Kansas, Oklahoma, and Missouri), seroprevalence in new regions and detection of BRBV in novel tick species warrant vigilance of this arbovirus as a public health concern.

## 4. Materials and Methods

### 4.1. Tick Vollection and Identification

**Figure 1:**
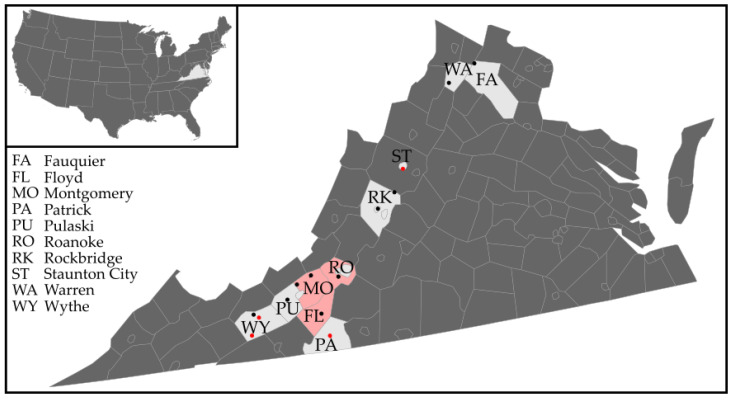
Map of study area in western Virginia. The black and red dots indicate the locations for each site sampled with established *H. longicornis* populations; red dots indicate sites where BRBV-positive ticks were detected. Counties shaded in pink had seropositive wildlife.

*Haemaphysalis longicornis* ticks were collected during routine tick surveillance at 13 sites across 10 counties in the western region of Virginia throughout the year of 2021 (Figure 1). These sites were selected based on previous surveys conducted in 2019 and 2020 where *H. longicornis* populations were detected [46]. Ticks were collected from vegetation using standard flagging methods using a 1 m by 1 m white canvas cloth attached to a dowel rod.

Ticks were collected and transported alive to the laboratory for immediate storage at −80 °C. Ticks were identified morphologically using keys [47] and a cold-chain maintained by using a chill table (Bioquip, Rancho Dominguez, CA, USA) positioned under a light microscope. Due to being a singular collection at an area where *H. longicornis* had not been confirmed before, the nymphal tick specimen collected in Patrick County was molecularly confirmed to species through sequencing of the 16S rRNA gene. The 16S rRNA PCR utilized the primers 16S+1 (5′-CTG CTC AAT GAT TTT TTA AAT TGC TGT GG-3′) and 16S−1 (5′-CCG GTC TGA ACT CAG ATC AAG T-3′), described in Black and Piesman [48] and de la Fuente et al. [49], in a 25 µL reaction using 12.5 µL of EconoTaq PLUS 2X Master Mix (Lucigen, LGC Biosearch Technologies, Madison, WI, USA), 2 µL of each primer at 10 µM concentration, and 5 µL of template. The PCR set up and thermocycler conditions were performed with modification from Black and Piesman [48].

### 4.2. Whole Nucleic Acid Extraction and Real-Time RT-PCR Detection of BRBV and HRTV

Both DNA and RNA were extracted from tick specimens using a whole nucleic acid extraction system developed by Crowder et al. [50], with DNA being sought for a separate study. Adult and nymphal ticks were cut in half bilaterally with one half going into the current extraction and the other half stored at −80 °C for later isolation attempts if needed. Larvae were kept whole for extractions. Ticks were grouped into pools based on the site where the ticks were collected, tick life stage, and collection date. In a dry homogenization step, ticks were removed from the cryofreezer and immediately pulverized (for 2 min at 30 hertz) individually, without diluent, in 2.0 mL bead beating tubes containing one 4.5 mm ball bearing and three 2.5 mm ball bearings using a TissueLyser II (Qiagen, Valencia, CA, USA). Buffer ATL (from the QIAamp MinElute Virus kit;Qiagen) was added to reconstitute each tick homogenate (up to 500 µL). An equal volume (in µL) of each tick sample was then combined to create pools to a total volume of 360 µL. Pending on how many ticks had been caught in the field, pool sizes varied from 1–20 nymphs and 1–8 adults per respective pool. Larvae were homogenized in pool sizes up to 200 larvae individuals in the same tube. Any remaining homogenate solutions were stored at −80 °C for confirmation assays. Following detection of virus-positive pools, viral RNA was extracted from individual ticks that comprised the pool using a QIAamp Viral RNA mini kit (Qiagen) as per manufacturer’s instructions. All samples were eluted in 50 µL of AVE Buffer.

Nucleic acid from each pool was initially screened for BRBV using a SYBR Green RT-PCR assay to amplify a portion of the PB1 (segment 2) gene using primers developed for this study (Table 4). The 20 µL reaction consisted of 10 µL of 2X iTaq Universal SYBR Green 1-Step Reaction Mix (Bio-Rad, Hercules, CA, USA), 0.25 µL iScript Reverse Transcriptase (Bio-Rad), 0.6 µL of each primer at 10 µM concentration, and 2 µL template. This reaction was performed on a Bio-Rad CFX96 Touch Real-Time PCR Detection System under the following conditions: 50 °C for 10 min, 95 °C for 1 min, followed by 40 cycles of 95 °C for 10 s, and 60 °C for 25 s. Potentially positive samples were determined by a C(q) value of less than 40 and a “normal” qPCR amplification curve shape. For each PCR assay, a positive control of RNA extracted from BRBV (Original strain; provided by BEI Resources) was used along with a no template control from each RNA extraction and a negative control of nuclease-free water.

Sample pools were also screened for HRTV using a probe-based RT-PCR assay developed by Savage et al. [26]. Briefly, we prepared a 50 µL reaction using reagents from a QuantiTect Probe RT-PCR kit (Qiagen) with 25 µL 2X Master Mix, 0.5 µL QuantiTect RT Mix, 2 µL of the forward and reverse primers (Table 5; 10 µM concentration), 1 µL of FAM-labeled probe (10 µM concentration), and 5 µL of template. The thermocycler conditions were a reverse transcriptase step of 50 °C for 30 min followed by 1 cycle of 95 °C for 10 min, then 45 cycles at 95 °C for 15 sec and 60 °C for 1 min. For each PCR assay, a positive control of RNA extracted from HRTV (MO-4 strain; provided by BEI Resources) was used along with a no-template control from each RNA extraction and a negative control of nuclease-free water.

### 4.3. Sequence Analysis

Virus-positive PCR amplicons were subjected to further confirmation using Sanger sequencing and compared to reported sequences of BRBV in NCBI GenBank (www.ncbi.nlm.nih.gov/genbank (accessed on 19 January 2022)) using the basic local alignment search tool (BLAST) [51]. We attempted to sequence-confirm each positive tick pool using two different amplicons: (1) 841 bp fragment of the PB1 (segment 2) gene and (2) 357 bp fragment of the PB2 (segment 1) gene (see Table 4). Amplicons were purified using a QIAquick PCR and Gel Cleanup Kit (Qiagen) and submitted to the Fralin Life Sciences Genomics Sequencing Center (Virginia Tech, Blacksburg, VA, USA) for Sanger sequencing in both directions. Raw sequences and chromatogram data were analyzed using Geneious Prime 2022.1.1 (Biomatter, Ltd., Auckland, New Zealand). Sequences were deposited in the GenBank database, with accession numbers as listed in the results section.

### 4.4. Bourbon Virus Isolation Attempt on Cell Culture

With the objective of viral isolation, 50 µL of a homogenate supernatant from each virus-positive tick sample were inoculated onto a confluent monolayer of Vero-76 cells (ATCC, Manassas, VA, USA) in 12-well culture plates (Genesee, El Cajon, CA, USA). Briefly, cryofrozen tick halves from each virus-positive tick pool were first dry-homogenized as described above, then suspended in 200 µL of tick diluent consisting of Dulbecco’s phosphate-buffered saline supplemented with 20% fetal bovine serum (FBS), 1% 100X solution of Penicillin-Streptomycin (P/S), and 1% amphotericin B solution at 250 µg/mL final concentration. Samples were incubated at 37°C with a 5% CO_2_ environment for 1 h, rocked every 10 min. Following adsorption, liquid overlay of 1 mL of Dulbecco’s Modified Eagles Medium (DMEM), supplemented with 2% FBS and 1% P/S, was added into each well. Samples were incubated at 37 °C and 5% CO_2_ for eight days and monitored daily for cytopathic effect (CPE) using an EVOS M5000 imaging system (ThermoFisher Sci, Bothell, WA, USA) microscope. Following this incubation, a 50 µL aliquot of each sample supernatant was transferred to a fresh Vero monolayer in a blind passage (even if not displaying CPE) and monitored for a further seven days. At the end of a second passage, RNA was extracted from the culture supernatant (regardless of whether CPE had been displayed) and tested as above.

### 4.5. Wildlife Serology

*Sample collection*. Local wildlife sera, collected as part of a separate project, were tested for evidence of exposure to Bourbon virus. Deer blood was collected in November 2021 (under Virginia Department of Wildlife Resources (DWR) permit #069946, VT IACUC#20-197), from the body cavity of 81 harvested deer brought to check stations for chronic wasting disease monitoring. Samples were obtained from Floyd (*n* = 33), Montgomery (*n* = 30), Giles (*n* = 1), and Pulaski (*n* = 17) counties, Virginia; these counties are adjacent to each other in southwest Virginia. In addition, blood was collected from *n* = 12 individuals from fresh roadkill and hunter donations in 2021 (IACUC#20-197, DWR salvage permit #069946), including 4 northern raccoons (*Procyon lotor*), 5 groundhogs (*Marmota monox*), 2 striped skunks (*Mephitis mephitis*), and 1 eastern cottontail (*Sylvilagus floridanus*), from Roanoke, Floyd, and Montgomery Counties. Blood was centrifuged at 5000 rpm for 6 min and subsequent sera transferred to a storage vial and heat inactivated using a bead bath (56 °C for 1 h) prior to analysis.

*Serology testing.* All sera were tested, initially at 1:20 dilution, by plaque reduction neutralization tests (PRNT), as described previously by Eastwood et al. [52], with slight modification, to determine antibody prevalence based on 80% neutralization (PRNT_80_) titers against BRBV. Serum was diluted in DMEM supplemented with 2% FBS and 1% P/S. Bourbon virus Original strain (provided via BEI Resources; passaged once in Vero cells to a stock titer of 5.9 × 10^5^ PFU/mL) was used in these challenge assays at a working concentration of 800 PFU/mL and as a virus-positive control (further diluted two-fold from 1:2 to 1:64); DMEM was used as a negative control; BRBV-positive rabbit antisera (provided by CDC) was used as a positive control. Briefly, 60 µL of serum and 60 µL of virus were challenged during 1 h incubation at 37 °C, and then, a volume of 50 µL was inoculated in duplicate into wells of a twelve-well culture plate. The modified method applied an overlay of 1.2% bactoagar (BD Difco, Franklin Lakes, NJ, USA) and 2X MEM (Gibco, Waltham, MA, USA) following the initial adsorption. This same overlay, later containing 1.5% Neutral Red solution (3.3 g/L; Sigma Aldrich, St Louis, MO, USA), was applied after 48 h incubation at 37 °C, then plaques were visualized after a further 24 h of incubation using a light box. Testing was performed at a BSL3 facility of Virginia Tech since BRBV is a risk group-3 pathogen [10]. End-point titration (serial two-fold dilution) of seropositive samples was conducted to establish the limit of antibody neutralization. Mean apparent seroprevalence values were calculated with 95% confidence limits (CI) for each species using a normal approximation for proportional data.

## Figures and Tables

**Table 1:** *Haemaphysalis longicornis* ticks collected in western Virginia in 2021.

Number of Collected *H. longicornis* Ticks							
County	Larvae	Nymphs	Females	Total Ticks	Total Pools	No. Pools Tested in This Study	No. Ticks Tested in This Study
Fauquier	7	64	1	72	7	7	72
Floyd	0	1	0	1	1	1	1
Montgomery	0	2	2	4	3	3	4
Patrick	0	1	0	1	1	1	1
Pulaski	157 ^1^	8	15	180	5	3	18
Rockbridge	18	15	0	33	5	5	33
Roanoke	282	0	0	282	2	2	282
Staunton City	5	10	0	15	2	2	15
Warren	3	15	0	18	2	2	18
Wythe	126	740	297	1163	80	8	192
Total	598	856	315	1769	108	34	636

^1^ These samples were stored at −20 °C prior to analysis and could not be used for viral RNA detection.

**Table 2:** C(q) results for BRBV-positive tick pools.

Sample Description			RT-PCR Screening ^a^		
			PB1 Gene (841 bp)	PB2 Gene (357 bp)	PB2 Gene (152 bp)
Sample ID	VA County	Pool Size and Life Stage (N = Nymph, L = Larvae, F = Adult Female)	C(q) ^b^	C(q)	C(q)
RH2-01	Patrick	1 N	38.2, 35.2	38.8	33.5
ST1-01	Staunton	10 N	38.6, 37.3	37.8	38.3
WY2-34	Wythe	125 L	39.4, 37.0	36.1	32.9
WY3-08	Wythe	10 F	38.2, 38.8	38.3	34.5

^a^ Assay developed in this paper. C(q) cut off at <39; ^b^ samples were run in duplicate.

**Table 3:** Sequence information for a portion of the PB2 gene of each BRBV-positive *H. longicornis* tick.

Sample ID	County	Tick Sample	Fragment Size	Genbank Accession No.	Comparative Genbank Accession No. (Query Coverage, Sequence Identity)
RH2-01	Patrick	Nymph	418 bp	ON153184	MT628410 ^1^ (100%, 99.9%)
					KU708253 ^2^ (100%, 99.9%)
ST1-01	Staunton	Nymph	345 bp	ON153185	MK453529 ^3^ (100%, 99.4%)
					MT628410 ^1^ (100%, 99.4%)
WY2-34	Wythe	Pool of 125 larvae	418 bp	ON153186	MT628410 ^1^ (100%, 100%)
					KU708253 ^2^ (100%, 100%)
WY3-08	Wythe	Female	405 bp	ON153187	MT628410 ^1^ (100%, 99.8%)
					KU708253 ^2^ (100%, 99.8%)

^1^ Genbank accession no. MT628410 (Dhori thogotovirus strain Bourbon virus). ^2^ Genbank accession no. KU708253 (Bourbon virus strain Original). ^3^ Genbank accession no. MK453529 (Bourbon virus isolate BRBV-STL2.

**Table 4:** Wildlife species showing neutralizing antibodies against BRBV, with county of origin within Virginia and end-point titer range of sera (at 80% plaque reduction) indicated.

County	Species	No. Individuals Sampled	No. Seropositive Samples	Sera Titer Range
Floyd	White-tailed deer	33	5	1:20–1:80
	Northern raccoon	1	0	-
	Striped skunk	1	0	-
Giles	White-tailed deer	1	0	-
Montgomery	White-tailed deer	30	4	1:20–1:160
	Striped skunk	1	0	-
	Eastern cottontail	1	0	-
Pulaski	White-tailed deer	17	0	-
Roanoke	Groundhog	5	3	1:40–1:80
	Northern raccoon	3	1	1:20

**Table 5:** Primer sets utilized in this study for BRBV and HRTV testing and tick species confirmation.

Gene Target	Amplicon Size	Primer Name	Primer Sequences (5′–3′)	Reference
PB1 (segment 2)	841 bp	PB1_F	CACCAAGAACATGTCTGAGCC	This study
		PB1_R	CTCAGTTCACCTGTAACCTCTGCC	
PB2 (segment 1)	357 bp	PB2_F	GTGCAARAGGGAGGTAGATATTGG	This study
		PB2_R	CTTTGAGTGATRAGYCCTCGGG	
PB2 (segment 1)	152 bp	PB2_Inner_F	CAGAATCCTTGATCGGGCCAG	This study
		PB2_Inner_R	GCATCCTATGGTGCTGAACTGTGG	
HRTV small (S) segment	86 bp	HRTV1-FOR HRTV1-REV HRTV1-Probe	TGCAGGCTGCTCATTTATTC CCTGTGGAAGAAACCTCTCC 56-FAM/CCTGACCTGTCTCGACTGCCCA /ZEN/-IBFQ	Savage et al., 2013
16S rRNA	454 bp	16S+1	CTGCTCAATGATTTTTTAAATTGCTGTGG	Black and Piesman, 1994
		16S−1	CCGGTCTGAACTCAGATCAAGT	
